# Role of the Ebola membrane in the protection conferred by the three-mAb cocktail MIL77

**DOI:** 10.1038/s41598-018-35964-6

**Published:** 2018-12-04

**Authors:** Ping Cao, Haihong Bai, Xinghe Wang, Jinjing Che

**Affiliations:** 1 Center for Drug Evaluation, CFDA, Beijing, People’s Republic of China; 2 Phase I Clinical Trial Center, Beijing Shijitan Hospital of Capital Medical University, Beijing, People’s Republic of China; 3 State Key Laboratory of Toxicology and Medical Countermeasures, Institute of Pharmacology and Toxicology, Beijing, People’s Republic of China

## Abstract

MIL77, which has a higher manufacturing capacity than ZMapp, comprises MIL77-1, MIL77-2, and MIL77-3. The mechanisms by which these antibodies inhibit glycoprotein are unclear. Infection by viruses with lipid-bilayer envelopes occurs via the fusion of the viral membrane with the membrane of the target cell. Therefore, the interaction between the antibodies and the EBOV membrane is crucial. We examined the interactions between MIL77 and the viral membrane using SPR. MIL77-1 selectively binds to viral membranes, while MIL77-2 and MIL77-3 do not. MIL77-1’s ability to screen the more rigid domains of the membranes results in a locally increased concentration of the drug at the fusion site. Although MIL77-2 recognizes an epitope of GP, it is not necessary in the MIL77 cocktail. These results highlight the importance of EBOV membrane interactions in improving the efficiency of a neutralizing antibody. Furthermore, the viral membrane may be an important target of antibodies against EBOV.

## Introduction

The Ebola virus (EBOV; species Zaire ebolavirus, family Filoviridae) is responsible for the 2014–2016 outbreak of Ebola virus disease (EVD) in West Africa^1,2^. Although extensive studies are actively investigating potential vaccines^3,4^ and therapies^5,6^, no prophylactic or post-infection therapy is currently approved for use against Ebola viruses. The EBOV surface glycoprotein (GP) is the key protein on the virion surface and is necessary and sufficient for infection^7,8^. GP is post-translationally cleaved by furin to yield the disulfide-linked GP1 and GP2 subunits^9^. The passive administration of antibodies targeting GP is among the most promising treatments for the often fatal consequences of EBOV infection^10,11^. After the optimization of different monoclonal antibody (mAbs) combinations, two antibodies from ZMAb (2G4 and 4G7) were combined with a mAb from MB-003 (13C6) to create the more potent cocktail ZMapp. ZMapp is currently the most promising antibody-based drug against EBOV and may soon be available after the completion of clinical trials^12^. However, attempts to manufacture ZMapp on a larger scale have been met with challenges partially due to low 4G7 yields in both plant and mammalian expression systems. To increase the manufacturing capacity for these mAbs, ZMapp-like mAbs were produced in modified Chinese hamster ovary (CHO) cells. This version of the cocktail is called MIL77 and is composed of MIL77-1 (containing the variable regions of c2G4), MIL77-2 (containing the variable regions of c4G7), and MIL77-3 (containing the variable regions of c13C6). MIL77 is a mAb cocktail against EBOV protected by China’s independent intellectual property rights^13^. MIL-77 was used to cure a British Ebola patient^14^. The framework regions of the antibodies in MIL77 were modified to be more similar to the human framework regions. The CHO cells used for the expression of MIL77 were engineered to prevent fucosylation, which is similar to the N-glycosylation present in plant-produced ZMapp; the absence of fucose increases the affinity of the mAbs for Fcγ receptor IIIa (FcγRIIIa) (CD16)^15^. Despite the importance of this mAb cocktail and its use as a therapeutic, the mechanisms by which these mAbs inhibit GP, including exactly how and where these mAbs bind GP, are not well characterized.

Infection by viruses with lipid-bilayer envelopes occurs via the fusion of the viral membrane with the membrane of the target cell^16^. Therefore, the role of EBOV membrane for the ability of these antibodies to prevent these viruses from entering cells is needed to been focus. The mechanism by which EBOV enters the host cells is similar to that of other type I viral membrane proteins, including HIV^17^. Our laboratory has studied the interaction between HIV fusion inhibitors and the viral membrane using surface plasmon resonance(SPR)techniques^18^. Our studies revealed that SPR is a powerful tool for the real-time monitoring of the steps involved in the mode of action of membrane-active peptides, including those that could not be previously detected using other techniques and are reported here for the first time^19^. Different interaction models can be used to fit SPR data, including one step and two step interaction models, membrane binding and membrane insertion models, electrostatic interaction models and hydrophobic interaction models^20^. The HIV viral membrane plays an important role in the fusion inhibition by locally increasing the concentration of the peptide at the fusion site. Therefore, the role of the Ebola membrane in the protection conferred by the three-mAb cocktailMIL77 was investigated using SPR in this paper. Our results are useful for an understanding of the mechanisms of the mAb cocktail against EBOV. Based on the role of the Ebola membrane in the protection from EBOV, we explained that the mechanism by which the efficacy of MIL77E (MIL77-1 + MIL77-3) is at least similar to that of MIL77^13^. This paper is the first to report the interaction between mAb cocktails against EBOV and viral membranes using SPR and the mechanism of action of MIL77 at the molecular level.

## Results

### Preparation of bilayer vesicle-coated sensor chips

SUVs (small unilamellar vesicles) with different rigid compositions and electric charges were prepared by extrusion through polycarbonate filters. The average diameters of the SUVs were approximately 80–90 nm as measured using DLS.

POPC, POPC:DPPC(1:1), DPPC, POPC:Chol(2:1), and POPG bilayers were absorbed onto L1 chips. The L1 chips were regenerated using 40-mM N-octyl β-D-glucopyranoside after each experiment, and the drift in the signal was less than 10 RU relative to the baseline before the experiment, indicating that the system was stable. The unsaturated phospholipid POPC was selected to mimic the ordinary eukaryotic plasma membrane, which consists of phospholipids containing saturated phospholipids. DPPC and Chol were selected as the EBOV viral membrane model.

### Binding affinity of MIL77 for the lipid bilayers as measured by SPR

Steady-state affinity model of MIL77 binding to membranes.

**Figure 1:**
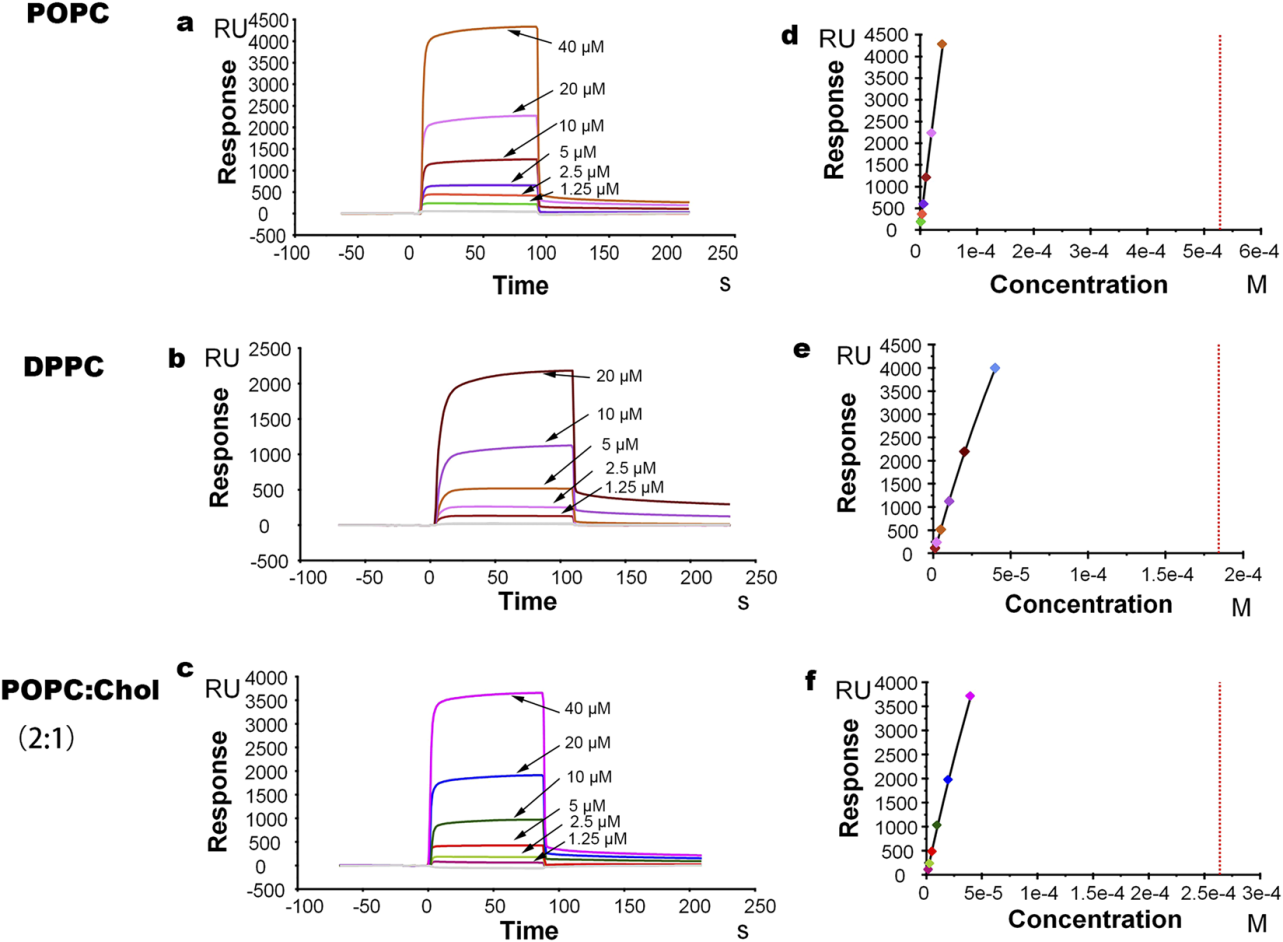
Panels (a,c). Representative sensograms of the binding between various concentrations of MIL77-1 and the lipid bilayer (L1 chip), POPC (panel a), DPPC(panel b), POPC/Chol(2:1w/w) (panel c). (Panels d–f) The corresponding relationships between the equilibrium binding response (RUeq) and the MIL77-1 concentration (C) (circles). The data were fit using the BIAcore’s steady-state affinity model(lines). MIL77-1 concentrations used are 1.25, 2.5, 5, 10, 20 and 40 μM.

POPC, POPC/DPPC (1:1w/w), DPPC, POPC/Chol(2:1w/w), and POPG bilayers were absorbed onto L1 chips. Representative sensograms of the binding of MIL77-1 to the bilayers are shown in Fig. 1.

The RU signal intensity increased as a function of the mAb concentration. Thus, the amount of mAb bound to the lipids is proportional to the increases in the mAb concentration.

**Figure 2:**
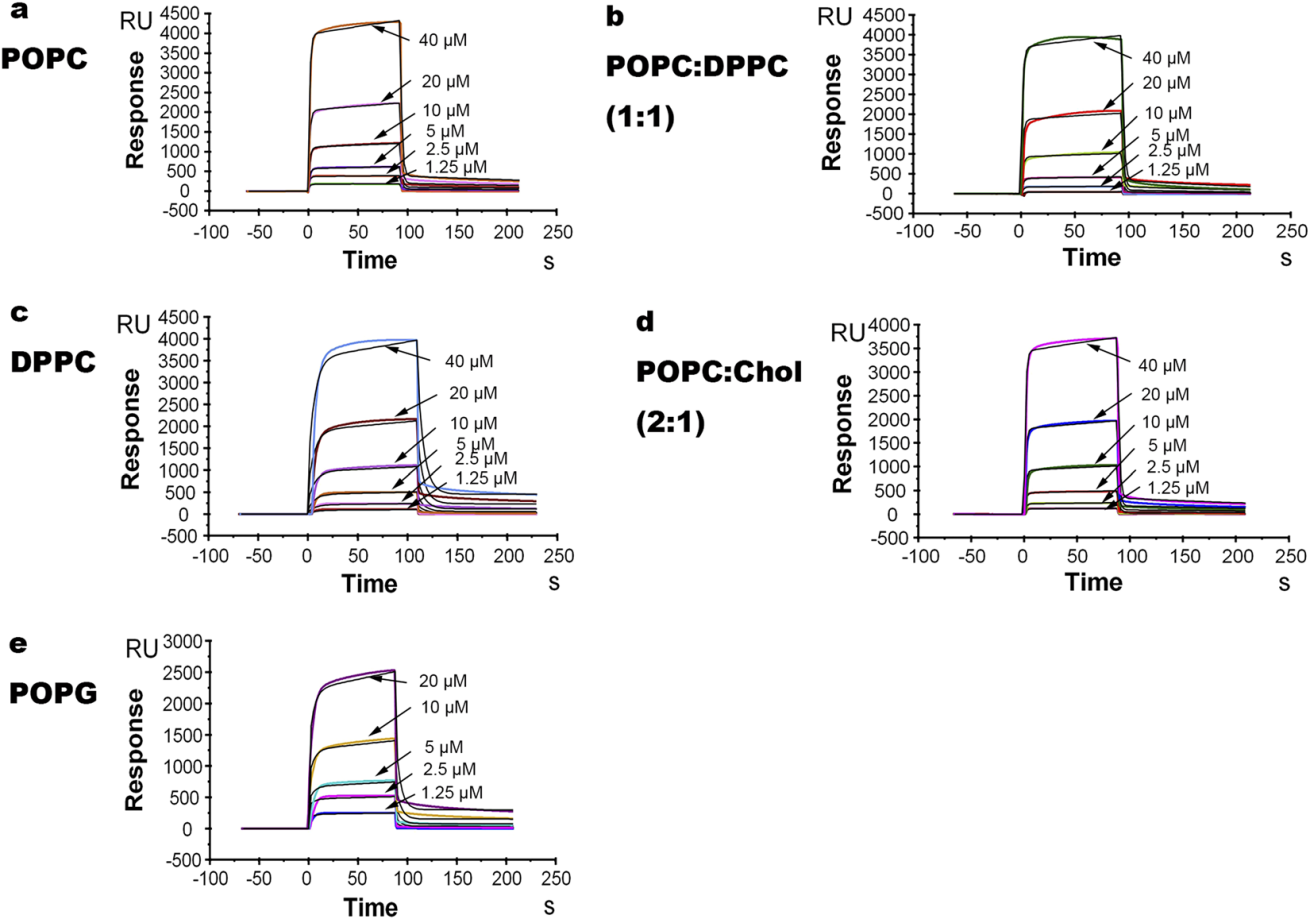
The two-state fitting model of the binding sensograms between various concentrations of MIL77-1 and the lipid bilayer (L1 chip), POPC (panel a), POPC/DPPC(1:1w/w) (panel b), DPPC(panel c), POPC/Chol(2:1w/w) (panel d), POPG (panel e). MIL77-1 concentrations used are 1.25, 2.5, 5, 10, 20 and 40 μM. Black curve is fitting result. Color curve is binding result.

Our system reached binding equilibrium during the injection of the sample; therefore, the affinity constants could be calculated from the relationship between the equilibrium binding response (Req) and the peptide’s concentration (C) using a steady-state affinity model (Fig. 2). Table 1 shows the affinity constants of MIL77-1, MIL77-2, and MIL77-3 to the bilayer membranes.

**Table 1:** Equilibrium Affinity Constants of the MIL77-1 with PC/Cholesterol and POPC, POPC:DPPC, DPPC, POPC:Chol, POPG Bilayers (L1 Chip) Derived According to a Steady-State Affinity Model.

Bilayer	MIL77-1 KD (10^−4^ M)	MIL77-2 KD (10^−4^ M)	MIL77-3 KD(10^−4^ M)
POPC	5.28 ± 3.00	2.09 ± 0.29	1.61 ± 0.38
POPC:DPPC(1:1)	1.88 ± 0.04	1.80 ± 0.38	1.85 ± 0.17
DPPC	1.84 ± 0.03	1.91 ± 0.32	2.30 ± 0.68
POPC:Chol(2:1)	2.64 ± 0.05	2.61 ± 0.48	5.69 ± 0.64
POPG	0.94 ± 0.07	1.28 ± 0.51	7.88 ± 0.45

The affinity of MIL77-1 for POPC/DPPC(1:1) is 3-fold higher than its affinity for POPC, which is similar to DPPC. The affinity of MIL77-1 for POPC/Chol(2:1) is 2-fold higher than its affinity for POPC. Thus, MIL77-1 has higher affinity for the POPC/DPPC(1:1), DPPC and POPC/Chol(2:1) bilayers than for the POPC bilayers. In contrast, the affinity of MIL77-2 and MIL77-3 for the bilayers is not affected by DPPC and Chol (the affinity of MIL77-3 for POPC:Chol(2:1) is 3.5-fold lower than its affinity for POPC). Because DPPC and Chol are rigid components of biomembranes, we can deduce that MIL77-1 selectively binds to rigid membranes, while MIL77-2 and MIL77-3 do not. The affinity of MIL77-1 for the negatively charged POPG membrane is 5.6-fold higher than its affinity for zwitterionic membranes. Thus, electrostatic interactions play an important role in the affinity of MIL77-1 for membranes. In contrast, the affinity of MIL77-2 and MIL77-3 for the bilayers is not affected by the charge of the phospholipid headgroup and only slightly differs between zwitterionic membranes and negatively charged phospholipid bilayers. Thus, the affinity of MIL77-2 and MIL77-3 for membranes is predominantly driven by hydrophobic interactions.

### Two-state model of MIL77-1 binding to membranes

Because MIL77-1 selectively binds to rigid membranes but MIL77-2 and MIL77-3 do not, we focused on the mechanism of the binding of MIL77-1 to membranes. We employed numerical integration analyses (BIAevaluation software, Biacore, Uppsala, Sweden) using a nonlinear analysis to fit an integrated rate equation directly to the sensograms^21^. While fitting the MIL77-1 sensograms globally (using different concentrations of MIL77-1) to the simplest 1:1 Langmuir binding model, a poor fit was obtained, confirming that this model does not represent the lipid binding mechanism of MIL77-1. However, an improved fit was obtained using a numerical integration of the two-state reaction model of the binding sensograms. This model reflects the following two-step process in which MIL77-1 interacts with lipid bilayers: the first step involves the actual binding, and the second step involves the insertion of MIL77-1 into the membrane. A set of sensograms with six different MIL77-1 concentrations was used to estimate the kinetic parameters. The average values of the rate constants obtained from the two-state model analysis are listed in Table 2. Data regarding the MIL77-1 and bilayer interactions are shown. The data reveal several important observations. The higher affinity of MIL77-1 for rigid membranes (i.e., POPC/DPPC, DPPC, and POPC/Chol) than its affinity for POPC membranes is due to the first step and dissociation of the second step. MIL77-1 binds ~10-fold faster (k_a1_) to and dissociates ~10-fold faster (k_d1_) from POPC/DPPC and DPPC than POPC in the first step and dissociates 20-40-fold faster (k_d2_) from POPC/DPPC and DPPC than POPC in the second step. Thus, MIL77-1 does not insert into rigid membranes. POPC/Chol is distinct. MIL77-1 binds ~10^5^-fold faster (k_a1_) and dissociates ~10^4^-fold faster (k_d1_) from POPC/Chol than POPC in the first step and dissociates 23-fold faster (k_d2_) from POPC/Chol than POPC in the second step. Thus, the major differences between the binding of MIL77-1 to rigid membranes and that to POPC membranes are observed during both the binding process and insertion process. Thus, MIL77-1 binds to rigid membrane mainly by surface binding rather than by insertion into the rigid membrane. The interaction between MIL77-1 and negatively charged phospholipid bilayers differs from its interaction with zwitterionic bilayers. MIL77-1 binds ~2-fold faster (k_a2_) and dissociates ~10-fold slower (k_d2_) from POPG than POPC in the second step, but the binding rate constants in POPG and POPC are similar in the first step. Thus, the major difference between the interaction with POPC and that with POPG is observed during the insertion process. MIL77-1 interacts with POPG by a partial insertion in the second step. The electrostatic interactions play an important role in the affinity of MIL77-1 for negatively charged membranes in the second step.

**Table 2:** Association (k_a1_, k_a2_) and Dissociation (k_d1_, k_d2_) Rate Constants in Bilayers Determined Using the Two-State Reaction Model.

	k_a1_ ± SE (×10^3^ 1/Ms)	k_d1_ ± SE (1/s)	k_a2_ ± SE (×10^−3^ 1/s)	k_d2_ ± SE (×10^−3^ 1/s)
POPC	0.232 ± 0.019	0.156 ± 0.006	1.517 ± 0.058	0.132 ± 0.055
POPC:DPPC (1:1)	3.997 ± 0.84	1.559 ± 0.14	1.446 ± 0.12	5.399 ± 0.84
DPPC	3.210 ± 0.34	1.913 ± 0.16	1.509 ± 0.083	2.725 ± 0.48
POPC:Chol (2:1)	34000 ± 15000	11900 ± 5200	1.315 ± 0.072	3.109 ± 0.51
POPG	0.111 ± 0.019	0.194 ± 0.014	3.461 ± 0.083	0.014 ± 0.17

## Discussion

The present study demonstrates that SPR is a powerful tool in investigating the real-time interactions between membrane active *mAb* and lipid bilayers that distinguishes among different mechanisms of action. H. Mozsolits^22^ reported L1 sensor chips can provides a membrane surface that mimics the physiological environment. It can be conveniently applied to the study of membrane-based biomolecular interactions and to measure the binding affinity related to these interactions. There have been a number of examples where these sensor chip have been applied to the study of peptide – and protein –membrane interactions. These applications range from the analysis of protein – protein and protein – ligand interactions in a membrane environment to the study of the direct binding of peptides and proteins to a specific phospholipid surface. SPR allows for the continuous monitoring of the following two major steps: the association of the mAb with the membrane, followed by a second step of insertion into the hydrophobic core or the inner surface of the bilayer.

Matteo Porotto *et al*. support the hypothesis that viral fusion occurs in confined areas in which the viral and host membranes interact^23^. Therefore, the viral membrane is a key factor for antiviral activity. The lipid composition of the viral membrane is strikingly different from that of the host cell membrane; the former is particularly enriched in cholesterol and saturated phosphocholine (DPPC)^24–27^. Cholesterol and DPPC are often laterally segregated in membrane microdomains or “lipid rafts” ^22,28^. Ebola virus buds from these lipid rafts^29^. Therefore, the unsaturated phospholipid POPC was selected to mimic the ordinary eukaryotic plasma membrane, which consists of phospholipids containing saturated phospholipids. DPPC and Chol were selected as the EBOV viral membrane model. Bilayer models were used to study the selectivity of the EBOV protective antibody for the viral membrane and the mechanisms of these interactions.

According to our results, the affinity of MIL77-1 for POPC/DPPC(1:1) is 3-fold higher than its affinity for POPC, which is similar to DPPC. The affinity of MIL77-1 for POPC/Chol(2:1) is 2-fold higher than its affinity for POPC. The affinity of MIL77-2 and MIL77-3 for the bilayers is not affected by DPPC or SM (sphingomyelin). The data show a marked difference in the interaction with the viral membrane between MIL77-1 and the other two MIL77 antibodies (MIL77-2 and MIL77-3). MIL77-1 selectively binds to rigid membranes (EBOV membrane), while MIL77-2 and MIL77-3 do not.

MIL77-1 contains the variable regions of c2G4, MIL77-2 contains the variable regions of c4G7, and MIL77-3 contains the variable regions of c13C6. c4G7 and c2G4 are neutralizing antibodies, whereas c13C6 is non-neutralizing but still provides *in vivo* protection from EBOV^30–32^. c13C6 binds to quaternary epitopes within a region in GP1 that is shared by GP and sGP^32,33^. c13C6 may facilitate immune responses, such as antibody-dependent cellular cytotoxicity (ADCC) or complement^34^. Therefore, the viral membrane may not participate in immune responses. The interaction between c13C6 and the viral membrane is not important for protection from EBOV. Therefore, the interaction between MIL77-3 and the viral membrane is not important for protection from EBOV. c2G4 and c4G7 are located near the GP1–GP2 interface. c4G7 and c2G4 could remain attached during the GP enzymatic processing. Their positioning allows these antibodies to prevent the structural rearrangements of GP required for viral fusion^34^. MIL77-1 selectively binds to rigid membranes, while MIL77-2 does not. According to our previous study^18^, most of the receptors are found on the rigid areas of the membranes. These findings may help explain the improved clinical efficiency of sifuvirtide, which locally increases the concentration of the peptide at the fusion site. Alam *et al*.^35^ propose that HIV neutralizing antibodies associate with the viral membrane in a required first step and are thereby poised to capture the transient gp41 fusion intermediate. Strong evidence supports the participation of the viral membrane in the neutralization of HIV-1. Thus, the viral lipid bilayer likely plays a role in the neutralization in addition to providing extra contacts for the antibody. Therefore, according to our results, the EBOV membrane also plays an important role by providing extra contacts for MIL77-1. We hypothesize that the molecular mechanism of action of MIL7-1 occurs on the EBOV target cell membrane and/or viral membrane. MIL77-1’s ability to screen the more rigid domains of the membrane provides a locally increased concentration of the drug at the fusion site. Therefore, we propose that the EBOV neutralizing antibodies of MIL77 act via a two-step mechanism. First, the antibodies attach to the viral membrane. GP1-GP2 maintains a native, prefusion conformation, and the membrane-bound antibody, which is concentrated on the virion surface, remains in rapid, reversible equilibrium with the antibody in solution. Once triggered, GP1-GP2 undergoes a cascade of conformational changes leading to the prehairpin intermediate, MIL77-1 becomes inserted into the target cell membrane and the transmembrane segment becomes anchored in the viral membrane. According lipid binding data, the hypothesis means that cell membranes are an MIL77 reservoir. The partition equilibrium tends to stabilize the MIL7-1 concentration in the aqueous environment when binding to GP occurs (i.e. improving inhibition efficiency). Moreover, the high levels of MIL77-1 accumulated at the cell surface render the interaction with other segments of GP possible if they reach the cell surface. The same hypothesis of HIV fusion inhibitor according only lipid binding date is reported^36^. Additionally, while MIL77-3 is not a neutralizing antibody, it provides protection from EBOV via immune responses.

Although initial studies suggested that c2G4 and c4G7 bind separate epitopes^36^, a more detailed structural analysis of ZMapp revealed that the two mAbs bind overlapping epitopes on the EBOV GP, suggesting that these two treatment components are redundant. Qiu *et al*.^13^ investigated whether the cocktail produced in the modified CHO cells had similar properties and comparable efficacy to the plant-produced ZMapp and evaluated the impact of removing the mAb c4G7 (MIL77-2). The results strongly suggest that the efficacy of MIL77E (MIL77-1 + MIL77-3) is at least similar to that of MIL77. MIL77-2 also had a much lower expression than MIL77-1 and MIL77-3 in their system. Therefore, a two-mAb cocktail also simplifies the production and approval processes and increases the safety profile of the treatment. According to our results, MIL77-2 can bind to the viral membrane selectively or non-selectively. Thus, the EBOV membrane can promote a locally increased concentration of MIL77-1 at the fusion site. MIL77-1 had a better anti-EBOV efficacy. MIL77-2 cannot selectively bind the EBOV membrane. Therefore, MIL77-2 cannot be enriched at the fusion site. Although MIL77-2 recognizes an epitope of GP, it is not necessary in the MIL77 cocktail of antibodies^13^. The antibodies that bound both GP and the viral membrane may have a good anti-EBOV efficacy. The viral membrane may be an important target of the anti-EBOV antibody.

The inhibition process must occur in extreme confinement between both the viral and the cellular membranes, therefore the interactions of fusion inhibitors with biomembranes are also important for determining their mode of action and activity. Previous studies^37^ have shown that c4G7 is shallow and nearly parallel to the viral membrane, whereas c2G4 is steep, with constant regions angling toward the viral membrane. Because of MIL77-1 containing the variable regions of c2G4 and MIL77-2 containing the variable regions of c4G7, the angle of MIL77-1 and MIL77-2 of approach to the viral membrane are also different. According to lipid binding data, the interaction of MIL77-1 and MIL77-2 and viral membranes are different. Whether the different binding angles confer differences in function is unclear. According to our results, the difference in the angle of approach to the viral membrane is maybe related to a difference in the interaction between the antibodies and viral membranes and differences in the anti-EBOV efficacy and mechanism of action.

Therefore, a suitable angle of approach and proximity to the viral membrane should be paid close attention for new effective neutralizing antibodies. This study provides a basis for the strategic selection of next-generation antibody cocktails against Ebola.

## Materials and Methods

### Materials

MIL77-1, MIL77-2, and MIL77-3 were gifts from Dr. Ming Lv from the Laboratory of Immunology, Institute of Basic Medical Sciences. 1-Palmitoyl-2-oleyl-sn-glycero-3-phosphocholine(POPC), 1, 2-dipalmitoyl-sn-glycero-3-phosphocholine (DPPC), 1-palmitoyl-2-oleoyl-sn-glycero- 3-phospho- (1′-rac- glycerol) (POPG) and cholesterol (Chol) were purchased from Avanti Polar-Lipids (Alabaster, AL, USA). N-octyl β-D-glucopyranoside and bovine serum albumin (BSA) were purchased from Sigma-Aldrich. HBS-N buffer (4-(2-hydroxyethyl)-1-piperazineethanesulfonic acid [HEPES] + NaCl), 0.2-M NaOH, L1 sensor chip and the Biacore Maintenance Kit were purchased from General Electric (CT, USA).

### Preparation of small unilamellar vesicles (SUVs)

SUVs comprising various components were prepared in HBS-N buffer. Briefly, dry lipids were separately dissolved in chloroform, and the solvents were evaporated using a rotary evaporator. The lipids were then resuspended in HBS-N buffer at a concentration of 0.5 mM with respect to the phospholipids. The resultant lipid suspensions were passed through a liposome extruder containing a 50-nm polycarbonate filter 19 times until a clear solution was obtained. SUVs of POPC, POPC:DPPC(1:1), DPPC, POPC:Chol(2:1), and POPG were prepared. The sizes of the obtained SUVs were measured by dynamic light scattering (DLS).

### Preparation of vesicle-coated sensor chips

The biosensor experiments were conducted using a BIAcore T200 (Biacore, Uppsala, Sweden) instrument with L1 sensor chips. The L1 chip contains hydrophobic aliphatic chains with exposed polar head groups; thus, as the chip contacts vesicles, a lipid bilayer forms^20^. We followed the protocol described in our previous study^18^.

Briefly, SUVs (80 μL, 0.5 mM) were applied to the L1 chip surface at a low flow rate of 2 μL/min. To remove any multi-lamellar structures from the lipid surface, NaOH (25 μL, 10 mM) was injected at a flow rate of 50 μL/min. BSA was then injected (10 μL, 0.1 mg/mL) to ensure the complete coverage of nonspecific binding sites. The bilayer of the L1 chip is linked to the chip surface and is used as a model membrane surface for studying mAb-membrane binding.

### Binding analysis using the SPR biosensor

The mAb solutions were prepared by dissolving MIL77-1, MIL77-2 and MIL77-3 in HBS-N buffer at concentrations ranging from 1.25 to 40 μM. The mAb solutions were injected over the lipid surface at a flow rate of 5 μL/min and then replaced by HBS-N buffer to allow mAb dissociation for 1000 s. The lipid bilayer was completely removed by injecting 40-mM N-octyl β-D-glucopyranoside, and each mAb injection was performed on a freshly generated lipid surface. All binding experiments were performed at 25 °C. A sensogram was obtained by plotting the response over time.

### Analysis of the SPR data

All data were evaluated using BIAevaluation software (GE). Our system reached binding equilibrium during the injection of the sample; therefore, the affinity constant could be calculated from the relationship between the equilibrium binding response (Req or RUmax) and the mAb concentration (C), using a steady-state affinity model. The rate constants of the dissociation (k_d)_ of the mAb from the phospholipid surfaces were determined by fitting the dissociation kinetics data to the following equation (1):

1 $${\rm{dR}}/{\rm{dt}}=-\,{{\rm{k}}}_{{\rm{d}}}\cdot {\rm{R}}\,$$

The rate constants of the association (ka) were determined using the following equation (2):

2 $${\rm{R}}={\rm{C}}\cdot {{\rm{K}}}_{{\rm{a}}}\cdot {{\rm{R}}}_{{\rm{\max }}}\cdot (1-{{\rm{e}}}^{-({\rm{C}}\cdot {{\rm{k}}}_{{\rm{a}}}+{{\rm{k}}}_{{\rm{d}}})\cdot {\rm{t}}})/({\rm{C}}\cdot {{\rm{k}}}_{{\rm{a}}}+{{\rm{k}}}_{{\rm{d}}})$$

where Rmax is the maximal binding capacity of the immobilized ligand surface expressed in RU, and C is the concentration of the mAb in solution. The values of the equilibrium dissociation constants (K_D_) were calculated as kd/ka. Because K_D_, which has the dimensions of the concentration, equals the concentration of free mAb at which half of the total phospholipid molecules are associated with the mAb, additional lines parallel to the y-axis were added to the figures to mark the location of the K_D_ value.

The sensogram of each mAb-lipid bilayer interaction was also analyzed by curve-fitting using a numerical integration analysis^38^. The BIAevaluation software offers different reaction models to perform complete kinetic analyses of the mAb sensograms. One curve-fitting algorithm (i.e., the two-state reaction model) was chosen. The data were fit globally by simultaneously fitting the mAb sensograms obtained at six different concentrations. The two-state reaction model was applied to each data set. This model describes two reaction steps^36^ that, in terms of mAb-lipid interaction, correspond to equation (3)

3 $${\rm{M}}+{\rm{L}}\begin{array}{c}{{\rm{k}}}_{{\rm{a}}1}\\ \leftrightarrow \\ {{\rm{k}}}_{{\rm{d}}1}\end{array}{\rm{ML}}\begin{array}{c}{{\rm{k}}}_{{\rm{a}}2}\\ \leftrightarrow \\ {{\rm{k}}}_{{\rm{d}}2}\end{array}{{\rm{ML}}}^{\ast }$$

where in the first step, mAb (M) binds to lipids (L) to yield ML, which is changed to ML* in the second step. ML* cannot dissociate directly to M + L and may correspond to the partial insertion of the mAb into the lipid bilayer. The corresponding differential rate equations of this reaction model are represented by equation (4)

4 $$\begin{array}{c}{{\rm{dRU}}}_{1}/{\rm{dt}}={{\rm{k}}}_{{\rm{a}}1}\times {{\rm{C}}}_{{\rm{A}}}\times ({{\rm{RU}}}_{{\rm{\max }}}-{{\rm{RU}}}_{1}-{{\rm{RU}}}_{2})-{{\rm{k}}}_{{\rm{d}}1}\times {{\rm{RU}}}_{1}+{{\rm{k}}}_{{\rm{d}}2}\times {{\rm{RU}}}_{2}\\ {{\rm{dRU}}}_{2}/{\rm{dt}}={{\rm{k}}}_{{\rm{a}}2}\times {{\rm{RU}}}_{1}-{{\rm{k}}}_{{\rm{d}}2}\times {{\rm{RU}}}_{2}\end{array}$$

where RU_1_ and RU_2_ are the response units for the first and second steps, respectively, C_A_ is the mAb concentration, RU_max_ is the maximal response unit (or equilibrium binding response), and k_a1_, k_d1_, k_a2_, and k_d2_ are the association and dissociation rate constants for the first and second steps, respectively.

## Conclusions

In conclusion, to dissert the molecular mechanisms by which the cocktail of neutralizing antibodies interact with EBOV at the membrane level, we examined the interactions between MIL77 and viral membrane models using SPR and demonstrated the importance of the membrane interactions in improving the efficiency of the neutralizing antibodies against EBOV. MIL77-1 selectively binds to viral membranes, while MIL77-2 and MIL77-3 do not. Thus, the EBOV membrane is an important participant in the binding and neutralization of EBOV by MIL77. MIL77-1’s ability to screen the more rigid domains of the membranes results in a locally increased concentration of the drug at the fusion site. Although MIL77-2 recognizes the epitope of GP, MIL77-2 is not a necessary component of MIL77. The antibodies that bound both GP and the viral membrane may have a good anti-EBOV efficacy. The efficacy of MIL77E (MIL77-1 + MIL77-3) is at least similar to that of MIL77. The viral membrane may be an important target of antibodies against EBOV. Additionally, a suitable angle of approach and proximity to the viral membrane may be required for new effective neutralizing antibodies. This paper is the first report the role of membranes in the mechanism of action of EBOV antibodies at the molecular level. In addition, this is the first study investigating antibody-membrane interactions using SPR. This study used feasible methods to study antibody-membrane interactions and elucidate the mechanism of action at the molecular level^37^.

## Electronic supplementary material

Supplementary Information
