# Supplementary material for: Role of the Ebola membrane in the protection conferred by the three-mAb cocktail MIL77
Source: Sci Rep. 2018 Dec 4;8:17628. doi: 10.1038/s41598-018-35964-6 (PMC6279787; doi:10.1038/s41598-018-35964-6)
Supplement: Supplementary file 1 — Supplementary Information [file 41598_2018_35964_MOESM1_ESM.pdf]

Role of the Ebola membrane in the protection conferred by the three-mAb cocktail MIL77

Cao Ping[1], Bai Haihong[2], Wang Xinghe[2*], Che Jinjing[3*]

[1]Center for Drug Evaluation, CFDA, Beijing, People's Republic of China

[2]Phase Ⅰ Clinical Trial Center, Beijing Shijitan Hospital of Capital Medical University, Beijing, People's Republic of China

[3]State Key Laboratory of Toxicology and Medical Countermeasures, Institute of Pharmacology and Toxicology, Beijing, People's Republic of China

*Corresponding author :

Che Jinjing, chejinjing80@126.com, 86-10-66930633

Wang Xinghe, wangxh@bjsjth.cn,86-10-68926401

These authors contributed equally to this work

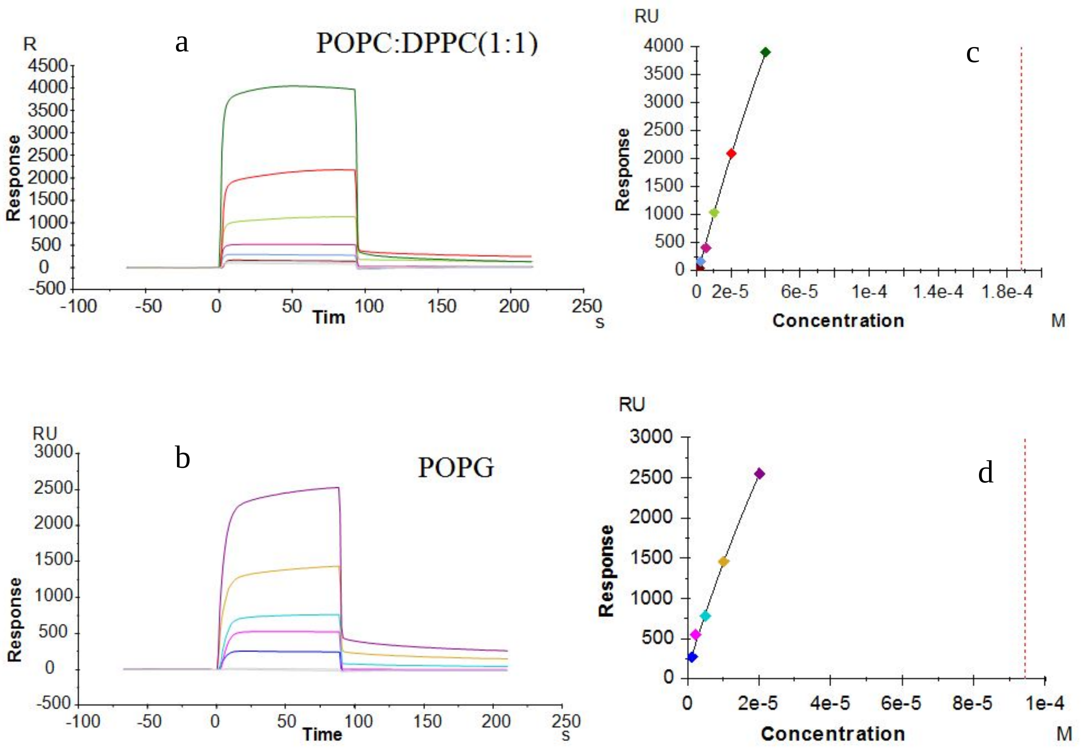

FIGURE S1:Panels a and b: Sensograms of the binding between various concentrations of MIL77-1 and the lipid bilayer (L1 chip), POPC/DPPC(1:1w/w) (panel a), POPG (panel b). Panels c,d: The corresponding relationships between the equilibrium binding response (RU_{eq}) and the MIL77-1 concentration (*C*) (circles). The data were fit using the BIAcore's steady-state affinity model(lines). MIL77-1 concentrations used are 1.25, 2.5, 5, 10,20 and 40 μM.

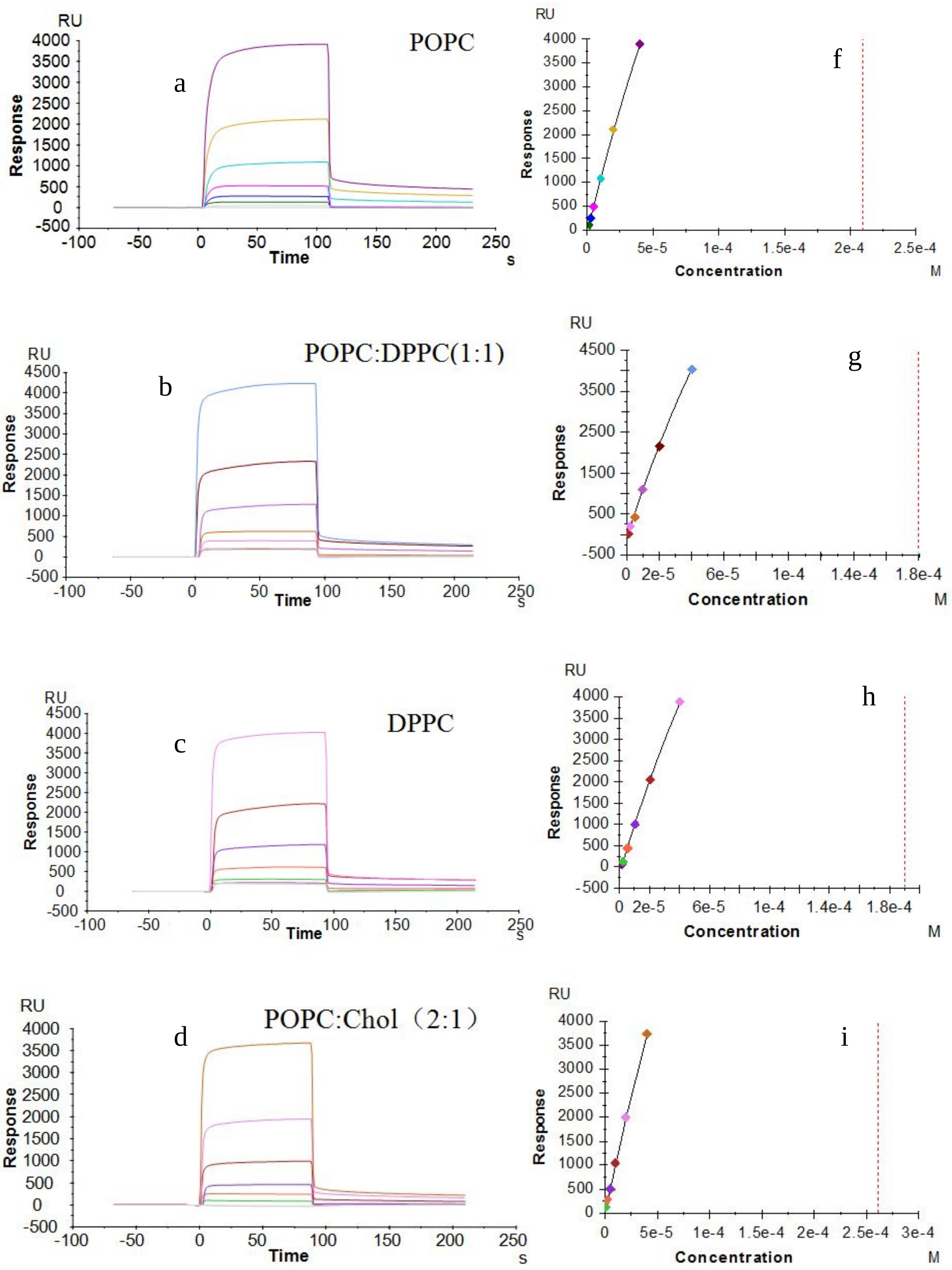

a
POPC
b
POPC:DPPC(1:1)
c
DPPC
d
POPC:Chol（2:1）
f
g
h
i
RU
Response
Time
s
Concentration
M

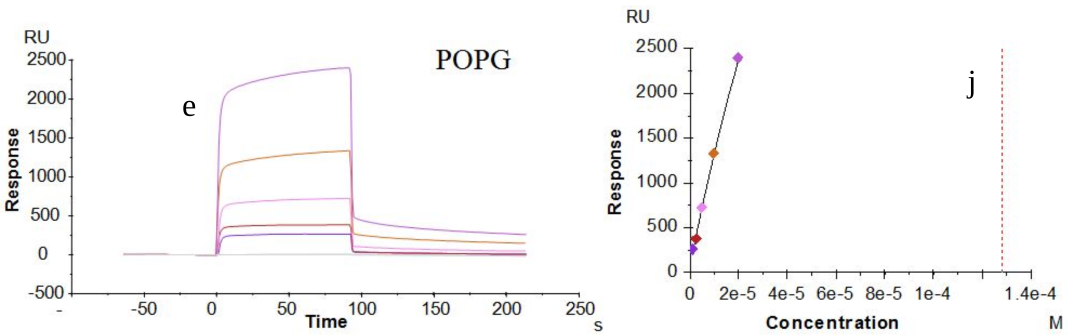

FIGURE S2:Panels a and d: Sensograms of the binding between various concentrations of MIL77-2 and the lipid bilayer (L1 chip), POPC (panel a), POPC/DPPC(1:1w/w) (panel b),DPPC(panel c),POPC/Chol(2:1w/w) (panel d), POPG (panel e). Panels f,g,h,i,j: The corresponding relationships between the equilibrium binding response (RU_{eq}) and the MIL77-2 concentration (*C*) (circles). The data were fit using the BIAcore's steady-state affinity model(lines). MIL77-2 concentrations used are 1.25, 2.5, 5, 10,20 and 40 μM.

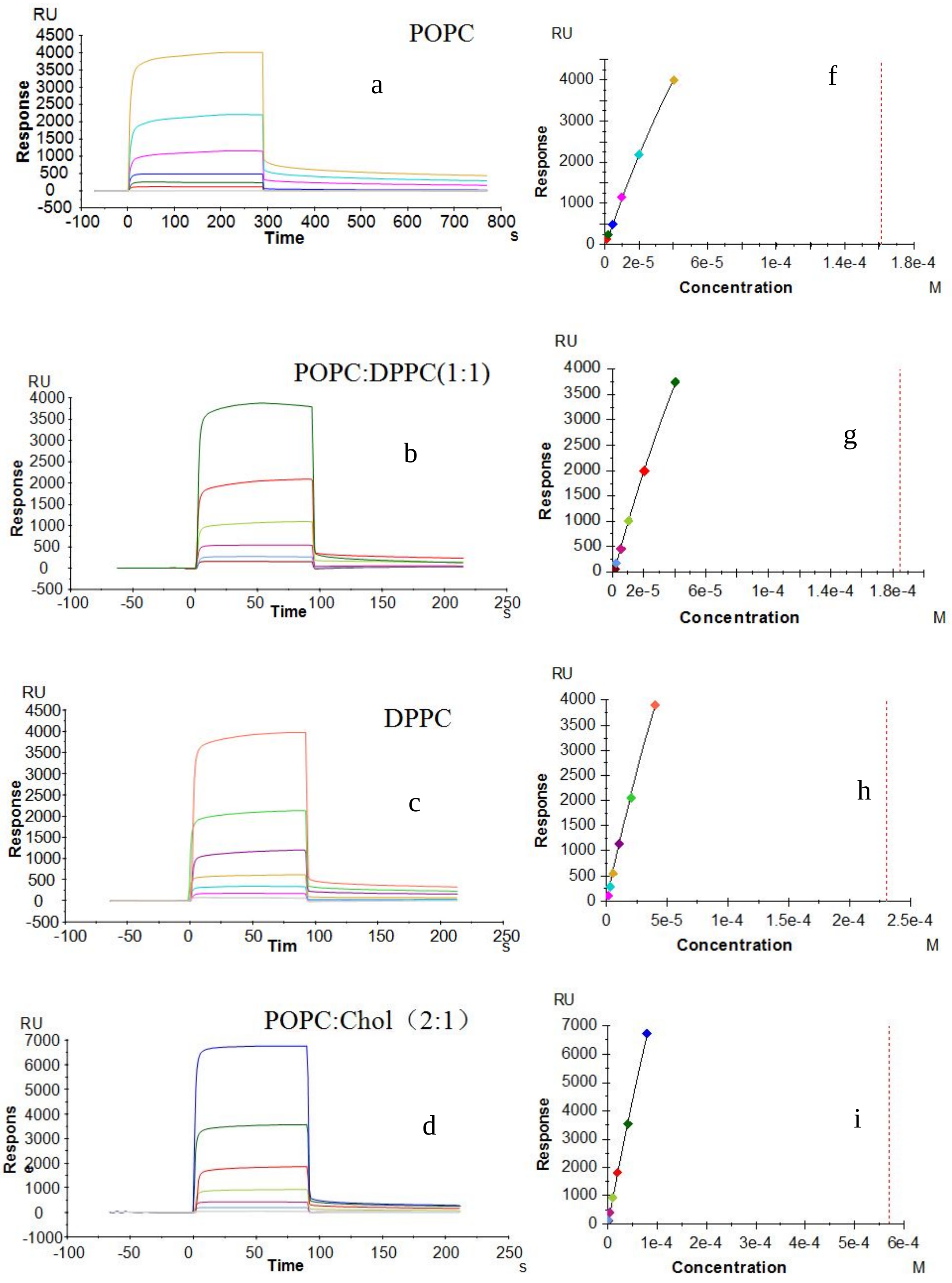
POPC
a
RU
Response
Time
s
f
Concentration
M
POPC:DPPC(1:1)
b
g
DPPC
c
h
POPC:Chol（2:1）
d
i

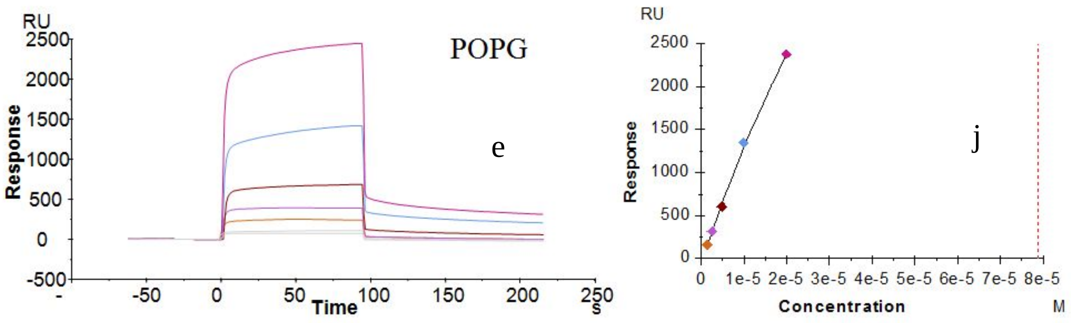

FIGURE S3:Panels A and D: Sensograms of the binding between various concentrations of MIL77-3 and the lipid bilayer (L1 chip), POPC (panel a), POPC/DPPC(1:1w/w) (panel b),DPPC(panel c),POPC/Chol(2:1w/w) (panel d), POPG (panel e). Panels f,g,h,i,j: The corresponding relationships between the equilibrium binding response (RU_{eq}) and the MIL77-3 concentration (*C*) (circles). The data were fit using the BIAcore's steady-state affinity model(lines). MIL77-3 concentrations used are 1.25, 2.5, 5, 10,20 and 40 μM.

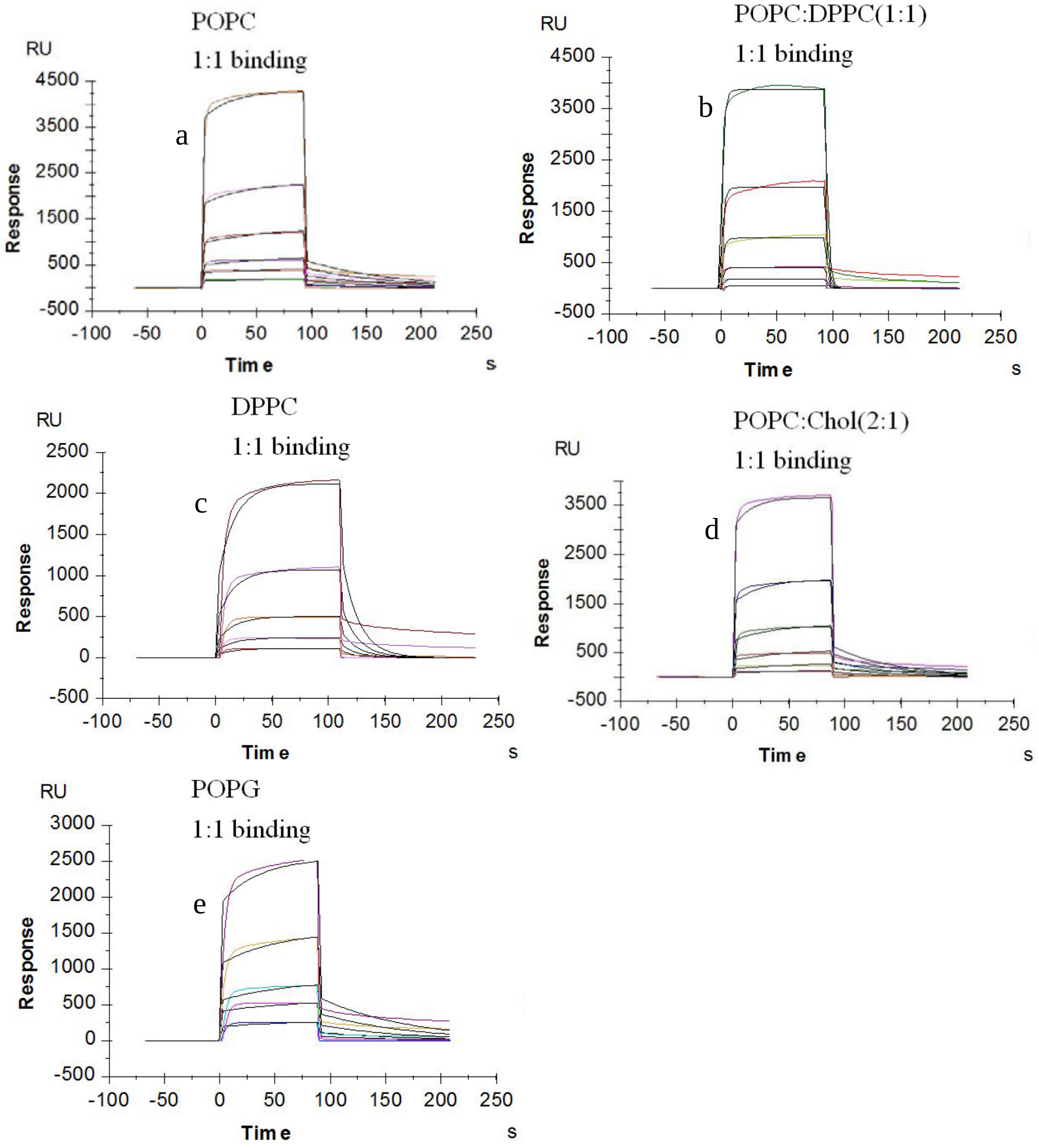

FIGURE S4: The one-state fitting model of the binding sensograms between various concentrations of MIL77-1 and the lipid bilayer (L1 chip), POPC (panel a), POPC/DPPC(1:1w/w) (panel b),DPPC(panel c),POPC/Chol(2:1w/w) (panel d), POPG (panel e). MIL77-1 concentrations used are 1.25, 2.5, 5, 10,20 and 40 μM. Black curve is fitting result. Color curve is binding result.
